# Assetization and the ‘new asset geographies’

**DOI:** 10.1177/20438206221130807

**Published:** 2022-11-08

**Authors:** Kean Birch, Callum Ward

**Affiliations:** 7991 York University, Canada; 8097 Uppsala University, Sweden

**Keywords:** Assetization, asset geographies, capitalization, financialization, rentiership, rentier capitalism

## Abstract

An asset is both a resource and property, in that it generates income streams with its sale price based on the capitalization of those revenues. Although an asset's income streams can be financially sliced up, aggregated, and speculated upon across highly diverse geographies, there still has to be something underpinning these financial operations. Something has to generate the income that a political economic actor can lay claim to through a property or other right, entailing a process of enclosure, rent extraction, property formation, and capitalization. Geographers and other social scientists are producing a growing literature illustrating the range of new (and old) asset classes created by capitalists in their search for revenue streams, for which we argue assetization is a necessary concept to focus on the moment of enclosure and rent extraction. It is a pressing task for human geographers to unpack the diverse and contingent ‘asset geographies’ entailed in this assetization process. As a middle range concept and empirical problematic, we argue that assetization is an important focal point for wider debates in human geography by focusing attention on the moment of enclosure, rent extraction, and material remaking of society which the making of a financial asset implies.

## Introduction: ‘New asset geographies’

It is often forgotten in discussion of financial capitalism that it is not all smoke and mirrors. There has to be something there to begin with. What we are seeing currently are attempts to make more ‘something’ there by producing new asset geographies (Leyshon and Thrift, 2007: 109).

Leyshon and Thrift (2007) argue that almost everything is being capitalized as an asset under financialized capitalism. An asset is both a resource, which generates incomes streams, and property, whose value is determined by capitalizing its future income streams and their relationship to broader political-economic trends (e.g. long-term rates of return). Leyshon and Thrift (2007) argue that while an asset's income streams can be bundled, aggregated, and then speculated upon, it is important to remember there is ‘something’ at the base of it all. Increasingly, they argue, a range of ‘new asset classes’ are emerging as capital/ists search for new revenue streams. A growing literature on the increasing societal scope and scale of this assetization process has emerged in human geography and cognate disciplines over the past few years, and we argue that understanding this process of assetization represents an important problematic necessitating engagement across, in particular, social studies of finance and critical political economy approaches. Such a dialogue positions us to understand asset formation as a process of ongoing enclosure based on economic rents which are dependent for valuation on future revenues.

An illustrative example of assetization is the transformation of the aerospace industry. Boeing no longer simply sells jet engines; rather, changing political-economic dynamics have led the aerospace manufacturer to adopt a ‘goods as service’ model whereby they lease engines to airlines which pay for them based on an hourly fee as part of tiered ‘CarePacts’ enabling a more lucrative maintenance service based on rent extraction rather than a one-time sale (Srnicek, 2016). Consequently, the economic value of the engines no longer derives primarily from it being a commodity whose exchange value is realized at point of sale, but from being a revenue-generating asset. While financialization, defined as the increasing dominance of finance and its logics (Aalbers, 2017), is part of this process, the concept does not capture the enclosure of resources or services in order to collect rents that are then capitalized as property. This creation of capitalizable property is deeply imbricated with finance but it is a distinct moment in the accumulation process.

Neither can this process be reduced to commodification: to commodify something is to make it exchangeable (Appadurai, 1986). Commodities are produced for sale, and as such their value is defined by the labour imbued in them as they are substitutable and subject to laws of competition. In resting on rent and enclosure without a particular orientation towards sale, assetization instead involves ‘the transformation of things into resources which generate income without a sale’ (Birch, 2015: 122). Rather than being based on competitive production in market conditions, asset formation is the creation of property that will afford a revenue stream – it is, therefore, the creation of rent-bearing property. A key point here is that assets depend on capitalization, being based on the long-term revenue streams that enclosure affords owners and that are capitalized with different discount rates reflecting different risk preferences and calculations. The market value of an asset depends on the estimated future rents it will afford, so for there to be a market for rent-bearing property the purchaser must borrow against future rent and capital gains. It is only after this capitalization that there is a viable market for tradable rent-bearing property and, therein, an asset. This introduces a subjective moment in which capital markets mediate the pulling of future value production into present circulation through particular, sociotechnically embedded, calculative practices (Mackenzie, 2009; Muniesa et al., 2017). This reification can be read in social constructivist (Birch and Muniesa, 2020) and pragmatist (Beckert, 2013, 2016) terms as ‘performativity’, as used in social studies of finance, or in Marxist value-theoretical terms as ‘real abstraction’ as imputed rents are realized in present circulation as fictitious capital (Harvey, 2006; Durand, 2017; Purcell et al., 2020). Something capable of generating rent is created through enclosure, then abstracted into an asset through capitalization of its future revenues, and this asset acts with material power on the present.

Examining the creation of assets as a socio-spatial process offers a common problematic centred on an underappreciated animating force of human geography today. Our aim is not to argue that assetization offers the means of a grand synthesis of different political-economic approaches. Rather, we seek to lay out the problematic of assets in contemporary capitalism from a geographical perspective in which, we argue, ‘assetization’ offers a middle ground concept cutting across approaches in articulating a common problematic for empirical enquiry on the issues of a rentier-dominated capitalism. The reification of fictitious capital and property is an area in which the line between social studies of finance and political economy approaches blur. It is in this space that we, as scholars who identify with different political economy traditions, argue that assets need to be empirically explored and theoretical distinctions clarified. We argue, therefore, that assetization is a useful meso-scale processual concept, which conceptually bridges macro-oriented notions of financialization and micro-oriented accounts of capitalization. In doing so, it focuses our attention on the details of abstracting capitalized property on the basis of rents while keeping in view the wider political economic currents and power relations this process sits in. 

Assetization has underpinned the shift towards modes of accumulation reliant on rent-bearing property rather than commodity production, as has been characteristic of the financialization of the economy since the 1970s (Pike and Pollard, 2010; Krippner, 2011; Ward and Swyngedouw, 2018; Aalbers, 2019a, 2019b), and the emergence of ‘rentiership’ and ‘rentier capitalism’ defined by asset ownership (Birch, 2017b; Christophers, 2019, 2020). Within this context, an increasing number of scholars from across various analytical and methodological schools and disciplines are turning their attention to this process (Birch, 2015, 2017a; Muniesa et al., 2017; Feher, 2018; Ward and Swyngedouw, 2018; Pistor, 2019; Birch and Muniesa, 2020; Bridge et al., 2020; Ouma, 2020a, 2020b; Strauss, 2020; Wu et al., 2020; Adkins et al., 2021; Langley, 2021; Fields, 2022). In articulating a central empirical problematic for understanding contemporary capitalism, we argue, this presents an opportunity for critical analysis within and across different approaches to political economy.

Focusing on the contextual details of the abstraction of assets is crucial for understanding how they embody and shape all manner of social conflicts over resources, and so has far reaching social and political implications. An asset entails not only a seemingly technical calculation and capitalization of future revenues, it also embodies and embeds a set of social and political assumptions about who gets to assert what social claims to those future revenues. The ‘asset condition’, as Muniesa et al. (2017) define it, represents the social and political logic that defines what asset investors should be able to secure from their (expected) investment returns over and above democratic or other political concerns. By focusing on how assets are made and constituted through post-politicalized governance (Ward and Swyngedouw, 2018), the concept of assetization allows us to centre the contingency of such sociopolitical relations as they are reified in the asset form (Langley, 2021). In specifying a distributed power relationship, assets are both outcomes and tools within class, gender, racial, and other social conflicts. Further analytical work on how different things are turned into assets and the resulting socio-spatial implications is thus necessary to scrutinize power relations across society. Human geographers are well-placed to undertake this critically important work.

In what follows, we first discuss how assets have been defined across the social sciences. On this basis, we then argue that there are three key topic areas in human geography to which the concept of assetization connects: financialization and the creation of new asset classes, globalization and shifting modes of governance, and inequality and the reworking of social reproduction. Subsequently, we suggest three future research agendas in human geography: (i) closer attention to the transformed spatio-temporalities wrought by assetization; (ii) further theoretical exploration of assetization as a process bridging micro and macro accounts of socio-spatial transformation, especially in providing some basis for empirically oriented middle ground theoretical insight between otherwise conflicting approaches to value and valuation; and (iii) understanding the increasing capitalization of everything as an asset, the contemporary ubiquity of rentiership, and the emergence of new asset classes and mechanisms of extraction (such as that of the platform economy). Finally, we emphasize that an understanding of assetization is necessary to the geographical critique of contemporary capitalist society, laying out commonalities in the empirical problematic this offers while pointing to the theoretical faultlines between approaches that require further debate and exploration.

## Studying assets and assetization: Social studies of finance and critical political economy perspectives

Assetization^1^ is an important empirical process that has been underspecified across human geography. The authors of this piece each identify with different traditions in political economy and have correspondingly different views on how an understanding of assets and assetization can be theoretically integrated with a critique of contemporary capitalism. Across approaches, however, formulating and specifying the concept of assetization centres attention on questions of distribution and the material impacts of calculative practices as a modality of systemic conflicts over resources. In this section, we sketch out the contours of the dialogue this requires by overviewing different treatments of assets as an object of analytical interest across the social sciences, focusing especially on social studies of finance and critical political economy traditions.

### Interrogating assets: The social practices of constructing future values

Classic institutional economists like Veblen (1908a, 1908b) and Commons (1924) provide some of the earliest analyses of assets as a distinct capitalizable resource (see Birch and Muniesa, 2020), emphasizing the legal and power dimensions of value derived from the ownership of ‘earning power’ (see Nitzan and Bichler, 2009; Dreyfuss and Frankel, 2015; Pistor, 2019; Kang, 2020). Notably, Veblen (1908a, 1908b) argued that ‘asset’ was a concept reflecting both ownership and valuation (Veblen, 1908b). And because an asset depends on ‘capitalizable value’ (Veblen, 1908a: 105), in the sense of representing the future and discounted earning potential, it centres investment as a key site of analysis (Levy, 2017). Veblen saw an asset's value, defined as its potential for capitalization, as an expression of ‘the extent of the control over the community that the asset secures’ (Gagnon, 2007: 596). More recent work in this Veblenian tradition by Nitzan and Bichler (2009) develops the specific concern with power with regards to the valuation of intangible assets.

A growing literature in science and technology studies (STS), and especially social studies of finance, builds on these institutional insights by unpacking the social contingency in how things are turned into assets. One particular focus has been the transformation of scientific knowledge into intangible assets, like intellectual property (IP) (Birch and Tyfield, 2013; Lezaun and Montgomery, 2015; Martin, 2015; Birch, 2017a, 2017b, 2020; Hogarth, 2017; Delvenne, 2021; Pinel, 2021). Other literature on the making of assets has focused on such diverse objects of analysis, as nuclear waste disposal to hospital beds (Muniesa et al., 2017), soybeans (Delvenne, 2021), and digital data (Birch et al., 2020; Birch et al., 2021; Geiger and Gross, 2021). Much of this research on assets hinges on a close examination of the social practices and everyday knowledges that go into the transformation of something into an asset (Birch and Muniesa, 2020), paying particular attention to the material devices (e.g. business plans) and calculative practices that enable assets to be valued (see Muniesa, 2012, 2014; Doganova, 2018). Characteristically, these studies draw on constructivist frameworks to dig empirically into the specific social practices that underpin financial valuation.

An important thinker in the social studies of finance is Eve Chiapello, whose work combining sociology and accounting has been pathbreaking in this regard. Notably, in her studies on financialization she differentiates between externalist accounts (e.g. ‘role and power of financial actors’) and internalist accounts (e.g. ‘socio-technical arrangements’) (Chiapello, 2019: 192–193). She focuses on the latter by examining the role of quantification and metrology in ‘making assets and liabilities’ and then ‘structuring monetary flows’, as well as the narratives that frame these more technical practices and devices. For example, ‘human capital’ is a narrative that redefines social practices as investments (e.g. education and learning). As Vollmer et al. (2009: 626) emphasize, such ‘financial cognition’ is an ‘achievement’ involving social practices and technical devices (MacKenzie, 2009; see Miller, 1998, 2008; Power, 2010). A key theme that emerges from this work is that financial value – or valuation, more accurately – is situated and constructed, reflecting different expert claims and practices about what counts towards a valuation and what does not. For example, Mennicken and Millo (2016: 15) point out that from 1977 to 1987 ‘the portion of goodwill in [acquisition] bidding rose from 1% to 44%’ reflecting a significant shift in the attitude towards a firm's intangible assets’.

Recent work by economic sociologists has picked this up to consider the impact and practices of asset valuation (Beckert, 2013, 2016; Boltanski and Esquerre, 2016; also Stark, 1996, 2009). For example, Beckert (2013: 229) is concerned with imagined futures in which, he notes, ‘shared expectations create demand for the asset’, meaning that pricing (and prices) should not be seen as an efficient process but rather as anchored in our collective belief systems. Consequently, Beckert (2016) challenges the very idea that we can even calculate the fundamental value of an asset using discounting practices. Rather, echoing Veblen and Chiapello, he emphasizes the collective constitution of (asset) valuations through shared expectations (also Elder-Vass, 2022). Similarly, in their theorization of ‘enrichment economies’ Boltanski and Esquerre (2016) argue that value is a collectively constituted practice, with the asset form a way of valuing things based on their expected prices. Finally, Stark (2009) elaborates a concept of ‘asset ambiguity’ as a contrast with Williamson's (1979) notion of asset specificity. Stark distinguishes an ‘ambiguous’ asset as one that can operate across different orders of worth, such that an asset can be a technological object (e.g. cellphone), financial object (e.g. sale unit), and cultural object (e.g. iconic brand), thereby enabling social actors to manage economic uncertainty through interdependent values (Stark, 1996).

The constructivist theoretical orientation of social studies of finance emphasizes the common belief systems that anchor financial valuations to expectations of future revenues that reflexively configure society itself (Birch, 2022). Such literature not only emphasizes the collective political-economic processes at play in the transformation of things into assets, but also the social expectations, relationships, and values on which the former depends. However, understanding the nature of this ‘asset condition’ also requires attention to the material practices and processes by which assets and made and maintained. This necessitates a critical political economy lens.

### Forming assets: Reshaping material economic practices

In contrast to the constructivist orientation of social studies of finance, critical political economy focuses on capital as an accumulation process, metamorphosing through distinct circuits: production in which a commodity is imbued with surplus value through labour; commodity circulation in which this value is realized through exchange; and a circuit of money capital mediating these transformations. Building on these insights, geographical political economy has its foundations in an insistence that the spatial circulation and distribution of capital is not epiphenomenal to production but integral to the economic system, with a particular focus on how urbanization has provided a spatial fix deferring crises in the primary circuit of production (Lefebvre, 1974; Harvey, 2006; see also Bok, 2018; Simpson, 2019). Central to this lies the theorization of economic rent, explaining how value is extracted through enclosure and circulates outside of production, in the process becoming a form of fictitious capital (see Harvey, 2006; Ward and Aalbers, 2016). This approach has centred, in part, on a long-standing debate around David Harvey's claim that there is an inherent tendency within capitalism to treat land as a financial asset, which entails land titles being treated as a form of interest-bearing capital and, subsequently, land use determined by maximal rental yield as opposed to use values (Haila, 2016).

Investigation of this concept of the ‘mobilization of land as a financial asset’ (Kaika and Ruggiero, 2015) has recently been extended to inform the analysis of assetization more broadly in explaining extractivist practices of specific capitalist actors (Fields, 2022; see also Ward and Swyngedouw, 2018; Ouma, 2020b). This is consistent with a growing focus on material practices of valuation in human geography more generally with Kay and Kenney-Lazar (2017), for example, calling for a focus on the plurality of valuation practices as a common agenda in political ecology. They argue that this would address a pressing need to theorize value in the context of finance and nature, as spheres that are central to contemporary accumulation but not traditionally considered productive of value. This is a call mirrored in Christophers’ (2018) claim that contemporary valuation practices require a retheorization of traditional Marxist approaches to value. Assetization here, then, is an important focus for understanding value in circulation through a pragmatic approach to material valuation practices, centring the discursive and practical operations of capital necessary to align diverse resources with money markets (Ouma, 2020a).

Those maintaining a value-theoretical approach, however, reject the conflation between valuation and value in its political-economic sense, insisting instead on the centrality of class struggle and the ‘value-rent nexus’ (Purcell et al., 2020; see also Baglioni et al., 2021), if we are to analyse how things such as natural resources acquire a price and circulate as fictitious capital. As Greco and Apostolopoulou (2020) argue, contra Kay and Kenney-Lazar (2017), natural resources are not productive of value but are given a commodity form (and hence a price valuation) based on the extraction of rents:

The fact that no abstract value is embodied in nature as such does not change the fact that the process of abstraction from the concrete heterogeneity of use values toward the ultimate equivalence of exchange value is a concrete, real process of abstraction (Greco and Apostolopoulou, 2020: 47).

The concept of real abstraction evokes readings of Marx that reject a substantialist view of value as labour in production and instead read in the concept of ‘socially necessary labour time’ as a relational theory centred on monetary circulation, combining subjective and objective elements (Pitts, 2021). The concept of real abstraction is central to this theoretical project, highlighting how abstractions emerge as the result of social interaction through the organization of the labour process and become ‘real’ in the sense of having practical power and autonomous dynamics not reducible to the labour process it emerged from (Sohn-Rethel, 1978; Postone, 1990; Toscano, 2008; Mann, 2018). Assetization, from this perspective, is the creation of exchange values that do not represent labour power but mechanisms of rent extraction circulating as capitalized real abstractions.

The question assetization poses in this critical tradition is how and by what means things are given this form of commodities – that is, exchangeable property – without being produced as such. Clearly, this is the outcome of various forms of class and social struggle both in the production process and, perhaps more prevalently, over ‘value-grabbing’ of rents in the sphere of distribution (Andreucci et al., 2017; Swyngedouw and Ward forthcoming). Here, the specific, power-laden contingencies of the way in which rents are abstracted into capitalized property is crucially important to current and future struggles over societal resources. The concept of assetization focuses attention on these contingent details, the ongoing enclosure they embody (Arboleda, 2017) and the subsequent class struggles over distribution as they are mediated by calculative practices (Andreucci et al., 2017).

Assetization prompts us to consider this material remaking of the world as a process of enclosure in which valuation practices depend upon future expectations (Birch, 2022). The different approaches we have considered here have fundamentally different understandings of value and the workings of the economy at the higher level of abstraction, but share a common problematic in trying to trace and theorize the formation of assets. Debate is needed here across different approaches as to the role and impact of valuation and its relationship to enclosures of value to extract rent if we are to respond to the challenge of explaining new asset geographies. In the next section, we highlight three key agendas pertaining to asset geographies that have emerged in human geography in recent years: financialization and asset creation, globalization and governance, and inequality and social reproduction.

## Engaging with new asset geographies

In the previous section, we overviewed the emergence of the asset form as a problematic in constructivist and critical political economy traditions, arguing that assetization offers a common empirical agenda facilitating meso-scale dialogue across these different analytical approaches. In this section, we build on this discussion to show how the problematic of assetization connects three central agendas in the geographical literature today: financialization, globalization, and social reproduction. These wide-ranging themes characterizing human geography highlight how assetization can help geographers to think across and within existing political-economic debates. Bringing assetization, as a contingent, contested process (Langley, 2021), into these debates opens up the everyday and systemic political-economic processes and practices of emerging asset geographies.

### Financialization and the creation of (new) asset classes

Financialization has become a key concept in human geography in the years following the 2007–2009 global financial crisis. Defined as the ‘increasing dominance of financial actors, markets, practices, measurements and narratives, at various scales, resulting in a structural transformation of economies, firms (including financial institutions), states and households’ (Aalbers, 2019a: 4), financialization positions finance as the object of study (Pike and Pollard, 2010), entailing an analysis of financial assets (e.g. derivatives), actors (e.g. investors), logics (e.g. discounting), and processes (e.g. valuation). More recently, financial geographers have started engaging with concepts of asset-formation where the impetus lies outside of, or is unevenly integrated with, capital market logics. For example, Aalbers (2019a, 2019b) centres on what he calls ‘financialization 2.0’ in relation to a rentier-dominated search for new asset classes (Wijburg et al., 2018). This brings assetization into the picture as part of a broader periodization of accumulation as capital responds to a low-growth environment by seeking new sources of capitalizable rents. In this sense, substantially theorizing the process of assetization is a necessary extension to financialization studies for the contemporary period.

Assetization helps us to more clearly conceptualize the restructuring of capital flows, urban spaces, social relations, and governance practices required to mobilize land as a financial asset (see Haila, 1991, 2016; Kaika and Ruggiero, 2015; Adisson, 2018; Ward and Swyngedouw, 2018). In particular, the assetization of land, real estate, and property relations through digital technologies adds a new dimension to these discussions, bringing together different political economy approaches (Fields, 2018, 2022). Key here are the ways that digital platforms (Srnicek, 2016; Langley and Leyshon, 2017; Sadowski, 2020; Atal, 2021) enable the intensification of assetization in the everyday forms of digital monetization, like Airbnb or Uber (Wachsmuth and Weisler, 2018). Platforms entail not just a new intensification of asset use and exploitation (e.g. of ‘idle assets’), they are premised on an ever more monopolistic transformation of resources into assets in which, as Fields (2018) shows, new techno-economic management of revenue streams creates new forms of financialized assets altogether. Fields (2022) stresses that technological devices and techniques have ‘fundamentally changed the way economic circulation is managed’, thereby enabling the streamlining of previously ‘lumpy’ and incommensurate revenue streams (e.g. housing rental income) to be smoothed out and so transformed into a liquid investment class for institutional investors. By utilizing an ‘operations of capital’ perspective (Mezzadra and Neilson, 2019), Fields links the micro focus of constructivist approaches on the performativity of financial devices and their everyday governance to the macro-oriented political economy critiques of capitalism.

The assetization of farmland highlights a similar remaking of agriculture and ecologies in line with the impulses of asset management (Li, 2014; Ducastel and Anseeuw, 2017; Sippel et al., 2017; Larder et al., 2018; Ouma, 2020a, 2020b). Here, Ouma (2020a, 2020b) illustrates how assetization entails moral struggles over the financialization of farmland. He argues that institutional investors increasingly treat land as a portfolio asset and thereby drive global agricultural land grabs, while seeking to offset these impacts through moral claims about the generation of ‘legitimate returns’ (see also Sayer, 2020). Alongside this transformation of farmland into an asset, it is evident that other environmental ‘resources’ are subject to specific forms of financialized extraction – like carbon trading (Felli, 2014), biodiversity offsets (Apostolopoulou et al., 2018), and carbon offsets (Bridge et al., 2020) – engendering intensified struggles over distributional resource inequities. Such environmental ‘value grabbing’ is a key mechanism through which economic rents are distributed to capitalist actors via the transformation and subsequent global circulation of natural resources as assets (Andreucci et al., 2017; Kay, 2017; Leitner and Sheppard, 2018). Notably, as Kay (2017) illustrates in her analysis of conservation projects in Maine, these mechanisms are also directed at preserving the environment (e.g. community forests), providing an example of how different social actors can intervene in the assetization process with very diverse political goals (see Langley, 2021). Assetization helps us to further unpack ongoing struggles over the transformation of social and environmental resources into the objects of financialized capitalism (Sayer, 2020).

Although there are clear linkages between assetization and financialization as concepts, the former does not necessarily imply the latter since the creation of an asset in the sense of giving exchangeable form to a rent-bearing property is fundamental to capitalist accumulation in general and does not imply a dominance of finance capital, actors, or logics (per Aalbers, 2017). Still, within a financialized economy, these two processes are closely entwined with each other, as financialization occurs in and through the capitalization of assets. In this, assetization can be seen as the supply side of financialization (Botzem and Dobusch, 2017; Ward and Swyngedouw, 2018), constituting the underlying resources as property and the capitalization of the subsequent income streams configured by financial innovations (Leyshon and Thrift, 2007). The central issue for assetization studies is how these resources and income streams are constructed and contested. An examination of the continuous search for and construction of new asset classes, therefore, positions assetization at the centre of debates in human geography about new forms of property (e.g. digital), markets, inequalities, and governance that analytically cut across and bring together specific, localized materialities with generic, globalized social relations (Aoyama et al., 2011; Aalbers, 2019a; Mezzadra and Neilson, 2019; Fields, 2022).

### Globalization and governance

The ongoing rescaling of the organization of socio-economic activity from the local to national to international scale (and back again) has been a central concern in human geography for some time (MacKinnon, 2011). The process of market and state rescaling has been shaped in part by an economy increasingly based on rentiership from investments in what Braun has termed ‘asset manager capitalism’ (2016: 263–265, inter alia). Asset-management at once requires close control at the level of the resource and their commensuration for global circulation, creating a dynamic of ‘glocalization’ (Swyngedouw, 2004; Torrance, 2009) in which the locus of economic governance falls onto sub-national geographical actors and international networks mediated through performative metrics. The nature and means of managing and governing assetization has thus been central to globalization and its attendant growth of finance.

The abstraction of exchangeable capital from spatially bound resources – what Harvey (1982, [2006]) terms the creation of capital liquidity from spatial fixity – has been central to accounts of uneven development in human geography (Harvey, 2006; see Gotham, 2012; Bok, 2018; Ward, 2021). In the process of financial liquidity creation, Pike and Pollard (2010) argue that localized material entanglements are contingently overcome to create a commensurable investment product. The resulting assets can then be traded anywhere, albeit often via global financial centres like London or New York (Van Meeteren and Bassens, 2016). For example, Pryke and Allen (2019: 1338) describe this process of asset abstraction in the case of a Californian water desalination plant:

For that [the infrastructure to become a financial asset] to happen the plant had to lose its ‘plant-like’ qualities and be assessed and parcelled out as part of an emergent asset class where its financial qualities were to the fore. It had to be ‘disassembled’, so to speak, broken down into its investment qualities, in order for it to move into the immaterial flows of international finance.

This process of decontextualization involved in this globalization of assets, however, raises questions over how to govern a reification that circulates separately from its embedded, material context (Savini and Aalbers, 2016). Some argue that relational proximity renders such assets governable in allowing for situated knowledge of assets through particular glocal sociotechnical assemblages and networked information exchange governable (Torrance, 2009; O’Brien and Pike, 2015; Pryke and Allen, 2019). However, the efficacy of this assetization of infrastructure cannot be assumed, and others contend that the reification involved in creating a globalized financial asset is itself a source of political-economic instability (Purcell et al., 2020; Ward, 2020). Assetization entails the transmission (or omission) of financial and legal knowledges as investment qualities. Unpacking how this affects the subsequent relation between the circulation of the financial asset and its underlying, very material revenue production is critical for geographers and evident in recent work on infrastructure (Adisson, 2018; Deruytter and Derudder, 2019; O’Brien et al., 2019; O’Neill, 2019). Understanding the making and circulation of assets is critical to understanding the governance of a globalized, financialized economy characterized by what Bryan et al. (2017) identify as ‘wealth chains’.

Understanding assetization in this way helps to explain how new forms of (asset) governance have facilitated international firms in seeking to acquire and manage material, local assets and thereby becoming ever more important partners in (sub-national) territorial governance (Harrison, 2014). This speaks to the notion of ‘seeing like a business’ that explores this relationship between city-regional governance and the regulation of global capitalism (Harrison, 2020), with a focus in particular on the financial and legal formation and management of local assets. For example, Adisson (2018) details the blurring of state and market boundaries underlying the shifting conception of French railways as financial assets whose redevelopment is premised on increasing capital gains (also see Adisson and Halbert, 2022 for Italy). As such, assetization problematizes the idea that local, embedded governance is necessarily more democratic or responsive to local needs. In contrast, asset management can be intensified through specific, yet multi-scalar, techno-economic, and legal means at the local scale. In this way, tracing the process of asset creation and management is necessary to unpack scalar tensions under conditions of financial globalization.

Simultaneously, this reworking of local asset-management practices corresponds with the deterritorialization of its circulation as an asset and the glocalization of the actors and institutions involved. Here, in particular, there is evidence of a distinct circulation of financial assets in global production networks (Coe et al., 2014; Grabher and van Tuijl, 2020), especially how asset abstraction has entailed distinctive networks and modes of governance as institutional investors seek to manage financial flows through ‘global wealth chains’ (Bryan et al., 2017; Seabrooke and Wigan, 2017). Echoing the growing ‘seeing like a business’ literature in economic geography (Brill and Robin, 2020; Harrison, 2020), Bryan et al. (2017) call for research to take the corporation itself as the scale of analysis in order to unpack the spatio-temporalities of the ‘wealth chains’ involved in constructing and managing assets (see Schwartz, 2016, 2017, 2022; Ward, 2021). Doing so in their study of offshoring and IP, Bryan et al. argue that trends in IP are:

…consistent with David Harvey's (1982) notion that property is increasingly being treated as if it were a financial asset. What seems to be happening in the contemporary role of IP and OFCs [offshore financial centres] is that intangible property is increasingly being produced, arranged and mobilised as an integrated industrial and financial asset (Bryan et al., 2017: 72).

At the same time, they argue, such financial assets are never pure (per Harvey) but hybrid in that they depend on specific legal frameworks and the offshore arbitrage therein, reflecting arguments made by Pistor (2019) and other socio-legal scholars on the legal dimensions of capitalism. This is fundamental to the geography of offshoring and property formation around IP assets, at the heart of which is regulatory and tax arbitrage (Clark et al., 2015; Bryan et al., 2017; Fernandez and Hendrikse, 2020), both within and across the new international division of labour advanced through globalization (Charnock and Starosta, 2016; Baglioni et al., 2021). Assetization, then, is necessary for understanding how governance is pursued on the ground, in the context of the ‘operations of capital’ (Mezzadra and Neilson, 2019) and its real abstractions (Purcell et al., 2020). As such, the formation of assets highlights the fundamental importance of geographically particular regulatory and legal mechanisms in what Kay and Tapp (2019) term ‘fiscal geography’. Here, they are referring to the ways in which the law guarantees and structures the circulation, expropriation, and smoothing of revenues from often very geographically diverse resources (also Blomley, 2019; Tapp, 2020). For example, in his analysis of oil storage assets, Simpson (2019) argues that the regulatory, legal, technical, and financial configuration of revenues leads to the ‘annihilation of time by space’ through the deliberate slowing down of this circulation of capital through geographical means (e.g. storing oil in railcars or oil tankers until its price increases). This transformation of a commodity like oil into an asset, as it is stored in railway cars or oil tankers, illustrates the need to understand the liminal spaces between commodity and asset, rent, and profit (also Braun, 2020; Delvenne, 2020). Here, then, assetization brings into view the commonalities underlying these disparate processes of property formation and regulation under conditions of financial globalization, and the attempts of investors to navigate this as both a political and an economic risk across diverse geographical dimensions (Raco et al., 2019; Brill and Robin, 2020). In so reshaping governance and regulation, the emergence of an asset economy is also fundamentally restructuring relations of social reproduction, linking to the final theme we identify.

### Inequality, welfare, and social reproduction

The long-term shift to wealth-based forms of social reproduction through assets have had profound effects on society more generally. Focusing on the Global North, Piketty (2014) argues that this shift to asset wealth over wages has exacerbated inequalities, creating new social and political dynamics with long-lasting and wide-ranging political ramifications. A societal divide has emerged between those who benefit considerably from capital gains on their assets, and those precariously positioned in a deteriorating labour market and welfare system. However, it should be noted that this divide is not a simple differentiation between wealthy rentiers and everyone else; it reflects broader and increasingly embedded expectations of asset-based social reproduction, especially through housing (Birch, 2015; Adkins et al., 2020, 2021; Wu et al., 2020), welfare (Watson, 2009; Doling and Ronald, 2010), education (Cooper, 2017; Komljenovic, 2020), and social life (Adkins, 2018; Williams, 2020). All of which is central to understanding the changing nature of class and other social struggles within contemporary capitalism (Adkins et al., 2020; Swyngedouw and Ward, forthcoming), and which has been extended to the Global South through what some scholars have termed ‘subordinate financialization’ (Powell, 2013; Leitner and Sheppard, 2018; Büdenbender and Aalbers, 2019).

As housing is the most accessible and significant asset for most people, its assetization has been fundamental to the changing welfare regimes, patterns of distribution, and class relations. The fostering of so-called ‘property-owning democracies’ and correspondent regimes of debt has been central to contemporary politics and policymaking (García-Lamarca and Kaika, 2016; Feliciantonio and Aalbers, 2018; August, 2020; O’Callaghan and McGuirk, 2021). The expansion of mortgage markets combined with denigration of universal social welfare provision has been a driver of asset-based welfare, especially in the erosion of universal pensions and their replacement by housing wealth to be tapped into upon retirement (see, inter alia, Watson, 2009; Doling and Ronald, 2010). Social reproduction generally is increasingly dependent on housing wealth, reconfiguring class and social relations around access to mortgage financing (Montgomerie and Büdenbender, 2015; Adkins et al., 2021), as both labour and housing markets have bifurcated. This bifurcation has not only impacted political-economic divisions (e.g. wealth holders vs. others), it has also reinforced gender divisions in households and families through the differential and gendered access to finance (Cooper, 2017; Adkins, 2018; Roberts and Zulfiqar, 2019), thereby further entrenching social inequalities as housing price growth outpaces income (Watson, 2009; Adkins et al., 2020, 2021).

Inequality of access to asset returns has had profound consequences as the extension of mortgage financing through liberalization and the related dramatic rise in value of housing assets has become an important means of demand stimulus across the Global North (Watson, 2009; Montgomerie and Büdenbender, 2015); a form of ‘privatized Keynesianism’ (Crouch, 2011). This has been an important element in the extension of cross-border finance to the Global South and other parts of the world as part of a dynamic of ‘subordinate financialization’ (Powell, 2013). Here, globally mobile capital from the financialized global core is absorbed in peripheral and other economies, with their real estate markets providing the prime asset (Leitner and Sheppard, 2018; Büdenbender and Aalbers, 2019). In China's post-socialist context, for example, Wu et al. (2020) point to the assetization of housing as the ‘Chinese way of financialization’, enabling the extension of financial logics, stimulation of demand, and their interpenetration with people's life courses through housing provision in a country that lacks the conventional indicators of financialization, such as a shift to market-based banking. The transformation of housing into an asset has thus been fundamental to processes of financialization, enabling, preceding, or, in some contexts, replacing the growth of a finance sector as part of the global restructuring of the division of labour (Charnock and Starosta, 2016).

The importance of housing and mortgage markets in the growth of finance reinforces Leyshon and Thrift's (2007) point that financial speculation alone is not sufficient: financialized capitalism also requires the underlying transformation of things into capitalized income streams (see also Wu et al., 2020). In recent years, following the bursting of the housing bubble with the global financial crisis, this search for assets has become more focused and specialized, with private equity companies and/or public–private partnerships (Birch and Siemiatycki, 2016) transforming the infrastructures of social reproduction in fundamental ways. For example, Gallagher (2021: 14) highlights how assetization of childcare in New Zealand has led to ‘wider societal implications’ through ‘deriving new forms of wealth from the crises of [child] care more generally’. Similarly, Horton (2021) and Strauss (2020) have investigated how the transformation of care homes into financial assets has impacted care labour in those workplaces, undermining both the quality of provision and labour conditions while providing a lucrative asset for investors to sweat. In this work, Strauss (2020) highlights the importance and necessity of incorporating gender, race, culture, and politics in the analysis of assets, emphasizing the need to analyse the normative and political dimensions of assetization alongside the political-economic (see also Ouma, 2020a).

## Unpacking the new asset geographies

Understanding the process of assetization opens up important avenues to explore contemporary capitalism and society. We have argued that this is a pressing empirical problematic which requires meso-scale investigations bridging (aspects of) constructivist-oriented social studies of finance and materialist critical political economy approaches, and in this section we propose three agendas for doing so in further research. Thinking broadly across human geography, these agendas are the importance of spatio-temporal specificity and contingency; the unpacking of the sociocultural dimensions of asset formation; and the contribution that assetization makes to a reinvigorated analysis of rentiership.

First, there is considerable room to investigate an emerging array of theoretical issues to do with the interactions between spatiality and temporality engendered by the diverse conditions, practices, and outcomes of assetization processes. A fundamental aspect of assetization is the need to understand the role played by space-time compression in the alignment of everyday knowledge claims and techno-economic practices (e.g. discounting) (Birch and Muniesa, 2020; Tellmann, 2020) with long-standing regimes and logics of capital accumulation and spatial fixity (Harvey, 2006). As Adkins et al., (2020: 17) put it, assets have ‘a particular temporal structure’ entailing investment in the present with the expectation of generating returns in the future. Consequently, temporality – especially the future expectations discussed by sociologists like Beckert (2013, 2016) – underpins assets in a number of ways that would benefit from further investigation.

Geographers are well placed to examine how distinct imagined futures are materialized in the present. Of particular relevance would be analyses of how future expectations about the highly contingent and collectively constituted yield of an asset are instituted in particular social relations, infrastructures, technologies, and built environments in the present, thereby locking-in specific political decisions. Or, conversely, analyses of how standardized, temporalizing calculations of risk that often underly the assetization process are disrupted by spatio-temporal uncertainties and tensions resulting from increasingly erratic political decisions (Ward, 2021; Vedel and Birch, 2020). For example, there are clear geographical implications resulting from political-economic logics and practices of depreciation and amortization that frame an asset's lifespan, including how societies organize welfare and social reproduction (Strauss, 2020), or how individuals understand their political and social agency (Feher, 2018). Notably, this would need to involve unpacking the ‘annihilation of time by space’ outlined by Simpson (2019); for example, aside from oil, other supply chains and asset classes across the world have slowed down during the COVID-19 pandemic. This entails a reconfiguration of the process of asset-formation and the expectations therein, as well as an intensifying reliance of asset markets on state intervention – another sense of annihilation of time by space (Ward, 2020). This transformation of spatio-temporalities wrought by the assetization process is central to geographical inquiry.

Second, assetization provides a distinct lens for human geographers who want to examine the contingent configuration of political economies through cultural and micro-scale narratives, knowledge claims, and social practices (Birch and Muniesa, 2020; Langley, 2021). While assetization is also necessarily macro-scale in outlook, concerned as it is with the transformation of capitalism, thinking geographically about assets provides an analytical linkage between micro phenomena, like capitalization practices (Muniesa et al., 2017), and macro phenomena, such as financialization (Aalbers, 2017; Fields, 2022). In this, assetization can provide a useful bridge between the macro-social focus on capital as an accumulation process whose logic exerts similar pressures on different social actors across diverse geographies, with the micro-social focus on a range of social, cultural, and political practices, techniques, values, and devices that engender geographically distinct and diverse asset forms and their consequences. As such, exploring asset geographies further would provide insight into the contingent geographical specificities of capitalism and society (Langley, 2021), as well as insight into how we might engage in a politics of contesting or repurposing assetization (Cumbers, 2012).

Finally, clarifying the role of assetization and asset geographies in contemporary capitalism is central to the growing critique of an accumulation regime dominated by rentiership (Zeller, 2008; Felli, 2014; Piketty, 2014; Haila, 2016; Andreucci et al., 2017; Birch, 2017b, 2020; Kay, 2017; Ward and Swyngedouw, 2018; Christophers, 2019, 2020; Strauss, 2020; Yrigoy, 2020; Langley, 2021). This is particularly the case with the rise of digital platforms that enable new forms of specifically digital rentiership (Srnicek, 2016; Langley and Leyshon, 2017; Birch et al., 2020; Sadowski, 2020; Birch and Cochrane, 2022; Fields, 2022). The resurgent interest in rentiership over the past decade provides an important lens through which to understand and contest the widening scope and scale of the transformation of an ever-growing range of things into assets from which to extract value. Such rentiership entails the control and/or ownership of resources and their revenues through socio-legal, technical, and spatial mechanisms; for example, rights to monopoly revenues (e.g. IP), emerging technologies (e.g. digital platforms), and claims to geographical uniqueness (e.g. geographical origins) (Pike, 2015; Birch, 2017a; Kay, 2017; Sadowski, 2020). Here, the increasing political importance of IP, digital platforms, intangible assets, and other techno-economic means of creating excludable goods and services are leading ever-more industries to transform their core productive activities and innovation strategies to centre upon the pursuit of, and reliance on, economic rents (Perzanowski and Schultz, 2016; Rikap, 2022; Schwartz, 2022).

In focusing on what Purcell et al. (2020) term the ‘value-rent-nexus’, assetization help further debates over value and valuation within human geography. It facilitates an approach to tracing out the mechanisms of extraction when it comes to the transformation of things into financial value (Mezzadra and Neilson, 2019; Ouma, 2020a, 2020b; Fields, 2022). From a value-theoretical perspective, meanwhile, the process can be understood as a form of ‘real abstraction’ (Sohn-Rethel, 1978; see Loftus, 2015; Greco and Apostolopoulou, 2020; Purcell et al., 2020), whereby ideational objects abstracted from practice are not epiphenomenal but have material impact upon the world. From this perspective, assets and their valuation are critical moments in contemporary class and social struggles, and ones that are crucial to understand as accumulation is extended ever more deeply into previously extra-economic phenomena (Andreucci et al., 2017; Kay and Kenney-Lazar, 2017). For those committed to the new reading of Marx in which value is read not as an imbibed substance of labour but a relational field verified in exchange, the dialogue between materialist and constructivist approaches which assetization opens up is crucial. As Pitts argues in his survey of value theory:

…because the commodified form of what is exchanged is at root a right to usage or ownership, rather than the simplistic appearance of the physical thing itself, those rights are contentious and contended inside and outside the operations of the market. For the institutionalists, this is principally a semiotic issue, but we could associate it more widely with social contestation about what should and should not be valued in monetary terms, or, where such terms are undisputed, struggles over the correct price to pay (Pitts, 2021: 78–79).

From this perspective, assetization is the problem that value-theoretical approaches must address in engaging with financialized capitalism. Assetization focuses our analytical lens on what Loftus (2015) identified as a lacuna in studies on the ‘violent geographies of abstraction’, and is particularly important for those engaged in a relational reading of Marx centred on abstraction as mediating between subjective and objective moments in the valorization process (see Pitts, 2021).

Assetization, as a concept, thus articulates the geographical, material, social, political, and cultural specificities of rentiership as a process of asset abstraction (Kay, 2017; Wang, 2020; Purcell et al., 2020). As the late Anne Haila (2016: 58) pointed out, present asset prices are ‘the claim for future rent’ where the conditions and causes of said rent are highly contingent. Rather than assuming that economic rents exist ab initio, assetization highlights the need to explore the techno-social processes through which rents are made and, consequently, how markets themselves are delineated and framed. Examining the social practices underlying this transformation of everything and anything into an asset helps open up several underlying assumptions in previous and current debates about rentiership. Assetization provides a focus for human geographers to examine how people come to frame something as a (potential) rent (Clark and Pissin, 2020), enclose and capture rental revenues, as well as the process of abstraction involved in capitalizing these income streams. Politically, unpacking rentiership and its class and social dynamics in this way helps to challenge the perceived dependence of societal prosperity and political stability on ever-rising asset values and the resulting tendency towards market concentration and monopoly.

## Conclusion

In this article, we argued that assetization specifies an empirical problematic connecting key themes across human geography. We made this argument from the starting point of Leyshon and Thrift's (2007) assertion that while almost everything is being capitalized within the contemporary financialized economy, there must be something underlying this capitalization: social relations, resources, institutions, and more being enclosed and (imputed future) rents being captured. Assetization specifies this process of ongoing rent extraction mediated by calculative practices as to future revenues in a way that is distinct from commodification and financialization. It requires a tracing out of the semiotics of valuation alongside analysis of the socio-material *something* underpinning valuation – of the value being extracted. In this, we argued, assetization requires further dialogue across approaches in human geography and the social sciences.

To this end, we highlighted a growing, cross-disciplinary focus on assets in a contemporary society dominated by the capitalization of (rentier) income streams. We argued that it is necessary to understand the restructuring of spatio-temporal relations involved in the process of creating and capitalizing these assets if we are to avoid treating finance as an abstract speculative process. On this basis, we then made our main argument that assetization, as a meso-scale concept specifying this empirical process of enclosing and capitalizing resources, connects key contemporary debates across human geography.

Most directly, we pointed to the growing concern with the formation of new asset classes in the financialization literature, arguing that this turn to assets in what Wijburg et al. (2018) refer to as ‘financialization 2.0’ remains theoretically underspecified in part due to the narrow teleological view that the otherwise useful concept of financialization can produce when applied at lower scales of analysis. We then argued that the concept of assetization offers important insights into the processes of globalization and concurrent rescaling of governance which has preoccupied human geographers. Notably, the need to control and manage social relations, resources, and institutions as assets has driven a rescaling to more networked forms of governance (Swyngedouw, 2004), often centred on wealth chains (Bryan et al., 2017; Pryke and Allen, 2019). Finally, we pointed to the literature on inequality and social reproduction which has increasingly centred on assets as scholars absorb Piketty's (2014) core insight that the tendency for return on capital to grow more than wages is a central driver of inequality (see Adkins et al., 2021). Here, we showed how growing inequality is compounded by modes of sociopolitical governance that centre on asset-based welfare (Watson, 2009; Doling and Ronald, 2010; Montgomerie and Buedenbender, 2015), underpinning contemporary social conflict and reproduction (Strauss, 2020; Gallagher, 2021; Horton, 2021). 

Our argument is that the empirical problematic the concept of assetization specifies requires a pluralistic dialogue across analytical perspectives. Our particular focus was on (constructivist-oriented) social studies of finance and (Marxist) critical political economy. Crucially here, assetization speaks to critical ongoing debates over the nature of value and valuation, to which the interaction of present rent extraction through contested enclosure and materialization of future expectations of that extraction into the present are central. Assetization does not promise a synthesis of theoretical frameworks in this, but a clarifying lens on an often murky debate that sharpens necessary distinctions. Further, from a value-theoretical perspective (Greco and Apostolopoulou, 2020; Purcell et al., 2020) assetization offers a link between these semiotics of power involved in capitalization and enclosure to the process of real abstraction, so centring the material contradictions of a financialized economy. Specifying assetization as a meso-scale concept thus opens space for dialogue in its empirical problematic, while spurring necessary clarification as to fundamental tensions around the conceptualization of the nature of value/operation of valuation across different approaches in human geography.

Our final section suggested three items towards a future agenda for human geographers to explore the ‘new asset geographies’ (Leyshon and Thrift, 2007). First, more work is needed on the spatio-temporal specificity and contingency of assetization (Simpson, 2019). An asset combines spatiality, for example, a material piece of land or an immaterial yet territorial claim on knowledge, with temporality, for example, an expectation of future incomes or legal limitation on changing future policy because of its potential impact on those future incomes (Dreyfuss and Frankel, 2015). Second, more work is needed to analyse the sociocultural dimensions of asset formation, ownership, valuation, politics, and so on. This might entail an investigation into the governance and governmentality of assets (Feher, 2018), to which analyses drawing on feminist, decolonial, antiracist, and Indigenous perspectives are much needed (Adkins, 2018; Strauss, 2020).

Finally, assetization is a crucial concept in the reinvigorated analysis of rentiership and rentier capitalism in human geography. Assetization helps us to think across existing geographical (and other) debates in this area, providing a means to bring together very different analytical perspectives and scholars who might not normally talk to one another. And there is a real urgency here, too. Clarifying the role of assets and assetization is key to addressing a range of emerging themes resulting from the current pandemic. Indeed, as many governments’ primary response to the social, economic, and political impacts of the COVID-19 crisis has been to support and reinflate asset values through massive central bank purchases of private debt and lowering of interest rates, it appears that part of the asset condition is being locked into a cycle of government-backed inflation of private assets followed by a socialization of the costs in a new round of asset inflation (Ward, 2020). In this context, understanding the creation and management of assets, as well as the everyday and societal distribution of costs and benefits they entail, is critical for envisioning a more equitable post-pandemic society.

## Notes

1. As a term, ‘assetization’ itself seems to appear first in the 1980s and 1990s in discussions of forestry policy and planning in China (Yigang, 1994). This reflects some of the earliest discussion of assets in the 19th century with the capitalization of forests by people like Martin Faustmann (Doganova, 2018). Similar arguments were made by neoclassical economists like Fisher (1907) who argued that the value of capital (i.e. asset) was constituted by the future (e.g. apple yield) (Muniesa, 2012). Subsequent discussion of assets in mainstream economics and management is evident in the work of ‘new institutional economics’ like Williamson (1979), who argued that firms are characterized by ‘asset specificity’, as well as resource-based approaches in management (Teece, 1986; Pisano, 1991).
